# GWAS of Diabetic Nephropathy: Is the GENIE out of the Bottle?

**DOI:** 10.1371/journal.pgen.1002989

**Published:** 2012-09-20

**Authors:** Carsten A. Böger, John R. Sedor

**Affiliations:** 1 Department of Internal Medicine II (Nephrology), University Medical Center Regensburg, Regensburg, Germany; 2 Department of Medicine and Department of Physiology and Biophysics, Case Western Reserve University and the Rammelkamp Center for Research and Education, MetroHealth System campus, Cleveland, Ohio, United States of America; Georgia Institute of Technology, United States of America

Diabetic nephropathy (DN) is associated with excess morbidity and mortality, in both type 1 (T1D) and type 2 (T2D) diabetic patients. Despite intensification of treatment, DN remains a growing problem worldwide [1]–[6]. In 2009, treatment of diabetic end stage renal disease patients accounted for approximately 40% of the US$43 billion expended for dialysis treatment in the United States

New management and treatment approaches are desperately needed and defining the genetic architecture regulating DN would accelerate their development. The landmark study by Seaquist et al. in 1989 [7] showed strong familial aggregation of DN and spurred the search for genetic risk variants associated with DN. However, family-based linkage and candidate gene analyses as well as the initial genome-wide association studies (GWAS), performed in single studies with limited power, showed inconsistent results in both T1D and T2D patients [8].

In this issue of *PLOS Genetics*, the GENIE consortium presents results of the largest DN GWAS meta-analysis performed to date. The discovery phase included 6,691 T1D patients from three cohorts, and SNPs with *p*<10^−5^ were moved forward into a replication analysis that included an additional 5,156 T1D patients in nine cohorts ascertained for nephropathy phenotypes [9]. Generally accepted phenotype definitions were used to identify DN cases (macroalbuminuria or end stage renal disease [ESRD] due to DN) and diabetic control individuals without nephropathy (diabetes duration of at least 10 years with normal albumin excretion). The combined metaanalysis for DN showed, disappointingly, no genome-wide signals, although an intronic SNP in *ERBB4* (chromosome 2) showed a consistent protective effect across cohorts (OR 0.66, *p* = 2.1×10^−7^). Intriguingly, *ERBB4* encodes a member of the EGF receptor tyrosine kinase family and modulates kidney tubule proliferation and polarity during nephrogenesis [10].

However, the DN definition essentially mixes two traits, each with distinct underlying pathomechanisms: ESRD as the extreme form of reduced kidney function (glomerular filtration rate, GFR), and macroalbuminuria reflecting severe glomerular filtration barrier dysfunction. Since these two traits have distinct genetic underpinnings [11]–[16], the authors refined their DN case definition to include only diabetic ESRD patients, which were contrasted with all other diabetic individuals regardless of albumin excretion level. Using these phenotypic criteria, the combined meta-analysis of discovery and replication cohorts identified genome-wide significant signals in an intron in the *AFF3* gene, and an intergenic locus between *RGMA* and *MCTP2* on chromosome 15. However, as the authors correctly point out, enthusiasm for *AFF3*, a transcriptional activator, should be tempered. This locus appears driven by two cohorts and technically did not replicate (*p* = 0.25 in stage 2 replication), although the effect direction was consistent across studies. The authors argue that power of the replication sample was limited for the alternative case definition due to the low number of ESRD cases (*n* = 363 versus *n* = 3,465 controls). The authors further support the association of *AFF3* with diabetic ESRD by providing experimental evidence that *AFF3* expression levels mediate TGF-β-1–driven fibrosis in an epithelial cell culture model. TGF-β-1 has consistently been implicated in the pathogenesis of fibrosis in DN, and these data provide a plausible function for *AFF3* in profibrotic pathways that characterize progressive diabetic kidney disease. However, the lack of significant association in replication analysis calls for independent confirmation of this locus in other studies before its implications for DN mechanisms can be drawn.

So—does this publication really let the “GENIE” for DN gene discovery out of the bottle, discovering at last *the* definitive “DN gene(s)”—or is this merely wishful thinking? It is sobering that this largest and long-awaited GWAS of T1D DN fails to provide unassailable statistical genetic evidence for associated variants, especially when compared to the success of GWAS in identifying convincing loci associated with other kidney diseases such as idiopathic membranous and IgA nephropathy, ANCA-associated nephropathy, or nondiabetic ESRD in African Americans [17]–[22].

**Figure 1:**
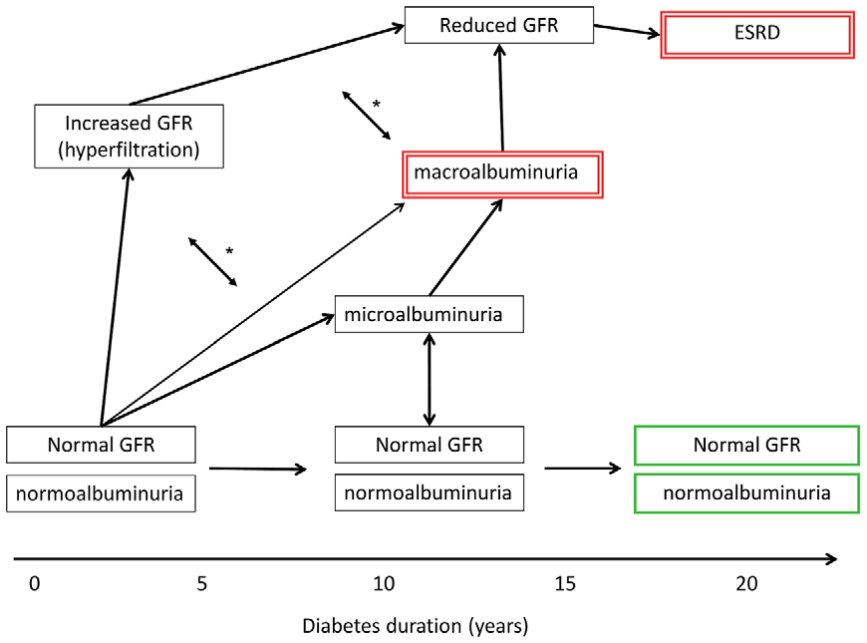
Schematic presentation of variable clinical courses of diabetic nephropathy. The case and control definitions used in the primary GWAS in Sandholm et al. [9] are indicated by green bold (controls) and red double-lined boxes (cases). GFR, glomerular filtration rate; ESRD, end stage renal disease. Microalbuminuria, urinary albumin excretion rate of 30–300 mg per day; macroalbuminuria, urinary albumin excretion rate of >300 mg per day. *, GFR and albuminuria may progress independently of each other, i.e., patients may have micro- or macroalbuminuria even though their GFR is normal or even slightly elevated. However, macroalbuminuria is usually associated with reduced GFR, and is a strong risk factor for progressive loss of eGFR and ESRD.

We believe that the definition of DN may lie at the crux of the overall disappointing reproducibility of genetic DN studies. In contrast to kidney diseases where diagnosis is based on a kidney biopsy (e.g., IgA nephropathy, membranous nephropathy) or imaging studies (e.g., ADPKD), the diagnosis of DN is almost always made using clinical criteria and not by histology. The clinical diagnosis uses phenotypic parameters derived from the typical course of DN: after many years of diabetes duration with normal GFR and absent albuminuria, DN onset is marked by mildly elevated albuminuria (also termed microalbuminuria), frequently with increased GFR (Figure 1). Subsequently, DN is characterised by overlapping stages of declining GFR and progressive proteinuria [23], finally leading to ESRD, with mortality as a competing risk. However, we have learned that proteinuria is more variable in DN than initially thought. In early stages, regression to normoalbuminuria is frequently observed [24]. Further, severe albuminuria (also termed macroalbuminuria) is not an invariate antecedent for profound kidney damage. Indeed, studies indicate that chronic kidney disease in diabetes may evolve in the absence of considerable proteinuria and progress to ESRD [25], justifying GENIE's analytic design contrasting diabetic patients with ESRD to all other diabetic subjects.

The present study consists primarily of cross-sectional studies and cannot capture the definitive DN outcome on an individual level. Using the most severe forms of DN to define cases reduces some potential misclassification but definitely does not overcome the critical inaccuracy of case definition not based on histology (many other kidney diseases can cause macroalbuminuria and ESRD). Further, the diabetic patient is exposed to many nonspecific kidney-damaging events in the course of disease (e.g., contrast agent imaging, nephrotoxic drugs, prerenal phases in infection and cardiovascular events), which in their sum also contribute to progression to ESRD. Overall, the sum of potential misclassification involved in using an exclusively clinical DN definition in cross-sectional studies reduces statistical power to detect underlying genetic variants.

With this meta-analysis of DN in T1D patients, Sandholm et al. have taken an important step towards defining the genetic architecture of DN. Strengths of the study include its large sample size, consideration of alternative DN phenotypes based on reproducible epidemiological and genetic data reported by other groups studying kidney diseases, and experimental support for the associated loci. Now the challenge will be building on these results. Two consortia, FIND and SUMMIT, should be reporting GWAS results for type 2 DN, and it will be interesting to see if common or unique genetic loci are identified of DN in T1D and T2D patients. Similarities in the clinical phenotypes and treatment responses of patients with T1D and T2D DN suggest a shared pathogenesis. Finally, additional meta-analyses of T1D and T2D DN cohorts with larger sample sizes, application of sequencing technologies, and use of more precise DN phenotypes from longitudinal studies should further define the genetic architecture of this most common cause of chronic kidney disease. These data will provide the foundation needed to advance understanding of diabetic nephropathy and impact patient outcomes.
